# A retrospective analysis of patients eligible for organ donation in adult intensive care units in Aotearoa New Zealand

**DOI:** 10.1177/0310057X251357317

**Published:** 2025-08-14

**Authors:** Lydia Shim, Cynthia J Wensley, Rachael L Parke

**Affiliations:** 1 Department of Critical Care Medicine, 58991 Auckland City Hospital, Te Toka Tumai Auckland, New Zealand; 2 School of Nursing, The University of Auckland, Te Toka Tumai Auckland, New Zealand; 3 Cardiothoracic and Vascular Intensive Care Unit, 58991 Auckland City Hospital, Te Toka Tumai Auckland, New Zealand

**Keywords:** Organ donation, brain death, Intensive Care Unit, New Zealand, circulatory death, outcome assessment, health care, retrospective studies, informed consent

## Abstract

To analyse characteristics of patients eligible for organ donation in New Zealand (NZ) Intensive Care Units (ICUs) and identify potentially modifiable factors that may benefit donation conversations and their outcomes. Design: A retrospective analysis of eligible patient data collected by Organ Donation New Zealand (ODNZ). Twenty-three adult ICUs in NZ from January 1, 2018, to December 31, 2021. Participants: Adult ICU patients eligible for organ donation via neurological determination of death (DNDD) or circulatory determination of death (DCDD). Patient and ICU characteristics, preparations for donation conversations, donation decisions, and reasons for non-approaches or declines were analysed. Descriptive statistics and binary logistic regression evaluated factors and outcomes. A total of 1,267 cases were analysed (DNDD = 687, DCDD = 580). Donation conversations occurred in 46.9% of cases, with 51.3% resulting in consent. Patients’ demographics and admission trends were similar to international reports. Male gender (p = 0.016) and ICU length of stay (p = 0.003) were associated with increased DCDD consent likelihood. Conditions such as encephalopathy (p = 0.012), and cardiovascular disease (p < 0.001) were associated with reduced donation conversation likelihood. Families of Māori patients were associated with reduced donation conversation likelihood (p = 0.002) and families of Māori (p < 0.001), Pasifika (p < 0.001), and Asian patients (p = 0.004) were associated with reduced consent likelihood. Early consultation with ODNZ and timely brain death confirmation positively impacted donation conversations and consent rates. Although not always practised, early ODNZ consultation and timely brain death confirmation were modifiable factors positively associated with conversations being approached and consent. Research exploring ICU staff and families’ perspectives may improve understanding of influencing factors

## Introduction

Facilitating organ donation is crucial to meeting the growing demand for life-saving transplants;^1–3^ however, patients meeting the eligibility criteria for donation are rare.^1^ Recognising potential donors, approaching families, and initiating donation conversations are responsibilities of intensive care unit (ICU) staff,^1,4,6^ and are key principles upheld by the New Zealand organ donation agency, Organ Donation New Zealand (ODNZ).^5,6^ In addition to ICU staff recognising eligible patients and approaching families to engage in donation conversations, organ donation also depends on families’ consent, influenced by the potential donor’s previously expressed wishes and their family’s wishes.^1,5,6^ In other countries, data are systematically collected and analysed on eligible donation cases.^7,8^ A study in the United Kingdom (UK)^11^ has explored modifiable and non-modifiable factors influencing consent rates. Similarly, studies in Canada^9^ and the United States (US)^10^ have identified modifiable and non-modifiable factors influencing conversation approaches as well as consent rates to establish best practices. NZ has 23 adult ICUs operating under an opt-in organ donation system, utilising donation by neurological determination of death (DNDD) and donation by circulatory determination of death (DCDD).^5,6^ ODNZ provides training for ICUs and has established ICU link teams that facilitate donation conversations, potential donor management, and routinely collect data into a central repository.^5,6^ However, these data have not been analysed to enhance understanding and inform organ donation processes. This study aimed to analyse existing data to understand the characteristics of patients eligible for donation in NZ ICUs, assess the rates of donation conversations initiated by ICU staff, and examine families’ decisions. Additionally, the study sought to identify potentially modifiable factors that may benefit conversation approaches and their outcomes.

## Methods

We conducted a retrospective analysis of eligible organ donation patients from 23 adult ICUs in NZ, between 1 January 2018 and 31 December 2021, from a central ODNZ data repository. Patient eligibility was determined using the following ODNZ criteria:^12^

Patients eligible for DNDD:

- no apparent brain reflexes AND
- fixed dilated pupils AND
- mechanically ventilated.

Patients eligible for DCDD:

- aged 70 years or less AND
- mechanically ventilated AND
- had withdrawal of curative treatment AND
- in whom death followed in less than 90 minutes.

### Data collection

Data obtained from the repository were:

Patient characteristics:

- age
- gender
- ethnicity
- year of death
- reason for admission
- ICU length of stay (LOS).

ICU data:

- ICU level (one = primary, two = secondary, or three =tertiary, as outlined by the College of Intensive Care Medicine of Australia and New Zealand)^13^
- DCDD accreditation status—indicates ICUs which have received ODNZ training to facilitate DCDD, but does not exclude other ICUs from pursuing DCDD.^5^

Preparation for donation conversations:

Whether ODNZ consultation was sought

- if brain death was formally assessed and confirmed
- method for confirming brain death.

Donation conversations and decisions:

- if a donation conversation was held with family
- outcome of conversation
- leader of the donation conversation (intensive care specialist, ICU nurse, anaesthetist)
- reasons for not holding a donation conversation
- reasons in which consent to donate was declined.

### Data management

Data were provided by ODNZ in de-identified Excel spreadsheets for all eligible DNDD and DCDD cases over the study period, and underwent data cleaning and categorising by researcher (LS) as follows:

- year of death: grouped into two categories: pre-COVID-19 and during COVID-19
- age: rounded down to whole numbers; stratified into five groups (0–20, 21–40, 41–60, 61–80, 80+ years)
- ICU (LOS): rounded down and categorised into three groups (0–3 days, 4–7 days, 8+ days)
- reason for admission: provided in three tiers: coded categories (natural, accident, suicide, homicide); clinical subcategories; and text descriptions were labelled into 15 categories (Supplemental File 1).

Cases meeting the criteria for both DNDD and DCDD were removed from the DCDD eligibility list for analysis to avoid duplicates.

### Data analysis

#### Descriptive statistics

Patient and ICU characteristics of eligible cases were analysed to determine proportions and minimum, maximum, median, and interquartile ranges for age. Incidences of donation conversations and family decisions were analysed separately for DNDD and DCDD, alongside healthcare professionals who led conversations. Documented reasons for not holding donation conversations were examined from most common to least, and families’ reasons for non-consent were also noted. All analyses were conducted using IBM SPSS version 29.0.2.0.

#### Binary logistic regression

Cases with documented reasons for not approaching donation conversations or declining donation were excluded. For cases without known reasons, binary logistic regression was conducted separately for DNDD and DCDD, to assess for relationships between variables and outcomes. Data quality checks were performed by the lead researcher (LS) and a hospital data analyst to ensure that there were no missing values and consistency in the data cleaning process. Variables: age, gender, ethnicity, year of death, reason for ICU admission, ICU LOS, ICU level, consultation with ODNZ, brain death confirmation (DNDD only), DCDD accreditation (DCDD only), and healthcare professionals who led conversations were added to the regression model by LS and a certified statistician step by step and evaluated for goodness of fit based on the Akaike information criterion and Bayesian information criterion.^14,15^ The variable ‘method for confirming brain death’ was excluded from the final model due to its irrelevance and skewed data. Categorical groups with sample sizes of less than 10 were excluded from the final model to maintain the integrity and validity of conclusions drawn from the analysis^14^ and included the following:

For the DNDD cohort:

- the ‘other' category within the ‘ethnicity' variable
- the ‘sepsis' category within the ‘reason for admission' variable.

For the DCDD cohort:

- The ‘homicide', ‘multi-organ failure', and ‘autoimmune disease' categories within the ‘reason for admission' variable.

Pairwise comparisons for multiple-category variables were conducted to evaluate reference points. Output measures included beta-coefficients, *P* values, odds ratios and 95% confidence intervals with statistical significance set at *P* < 0.05.

### Ethics approval

The study was approved by the Auckland Health Research Ethics Committee on 7 November 2022 (ref. AH24963).

## Results

### Characteristics of patients and ICUs

In total, 1267 eligible donation cases were analysed, half of which were between 41 and 63 years old, with more men than women (58.6% vs. 41.4%, respectively) (Table 1). Most eligible patients were NZ European, followed by Māori, Pasifika, and Asian. The leading reason for ICU admission was brain haemorrhage (371/1267, 29.3%). No significant differences were seen between eligible DNDD and DCDD cases regarding age, gender, ethnicity or before/during the COVID-19 pandemic. (Table 1). Overall, 1007 (79.5%) patients were admitted into level three ICUs, 197 (15.5%) into level two, and 63 (5.0%) into level one, with no notable differences in distribution between eligibility for DNDD and DCDD. Additionally, 479/580 (82.6%) of eligible DCDD cases occurred in DCDD-accredited hospitals.

**Table 1.** Patient characteristics of eligible donation cases.

Variables	All patients (*n* = 1267)	DNDD (*n* = 687)	DCDD (*n* = 580)
Age, years	Minimum: 15	15	15
	Maximum: 90	90	69
	Median: 55	50	58
	Interquartile range: 41–63	35–63	48–64
Gender, *n* (%)	Men: 742 (58.6%)	377	365
	Women: 525 (41.4%)	310	215
Ethnicity, *n* (%)	NZ European: 689 (54.4%)	365	324
	Māori: 329 (26.0%)	169	160
	Pasifika: 119 (9.4%)	71	48
	Asian: 109 (8.6%)	73	36
	Other: 21 (1.7%)	9	12
Year of death, *n* (%)	Before COVID-19: 663 (52.3%)	346	317
	During COVID-19: 604 (47.7%)	341	263
Reason for admission, *n* (%)	Brain haemorrhage: 371 (29.3%)	294	77
	Cardiac arrest: 152 (12.0%)	74	78
	Brain ischaemia: 116 (9.3%)	80	36
	Sepsis: 112 (8.8%)	3	109
	Cardiac failure/cardiovascular disease:		
	102 (8.1%)	24	78
	Suicide: 102 (8.1%)	63	39
	Road trauma: 81 (6.4%)	62	19
	Other trauma: 61 (4.8%)	33	28
	Encephalopathy: 49 (3.6%)	28	21
	Gastrointestinal failure: 34 (2.7%)	0	34
	Respiratory failure: 32 (2.5%)	0	32
	Cancer: 24 (1.9%)	12	12
	Homicide: 19 (1.5%)	14	5
	Multi-organ failure: 8 (0.6%)	0	8
	Autoimmune disease: 4 (0.3%)	0	4
Intensive care unit length of stay, *n* (%)	0–3 days: 1003 (79.2%)	604	399
	4–7 days: 152 (12.0%)	59	93
	8+ days: 112 (8.8%)	24	88

Values are *n* (%) or median (interquartile range).

DNDD, donation by neurological determination of death; DCDD, donation by circulatory determination of death.

### Preparation to approach donation conversations

Of the 1267 eligible donation cases, 597 (47.1%) were consulted with ODNZ, including 63.8% of DNDD cases (438/687) and 27.4% of DCDD cases (159/580). Of the 670 (52.9%) cases not consulted, 36.2% (249/687) were potential DNDD cases, and 72.6% (421/580) were potential DCDD cases. For eligible DNDD cases, brain death was confirmed in 53.0% (364/687), primarily using an assessment-by-two-clinicians method (332/364, 91.2%).

### Donation conversations and decisions

**Figure 1.**
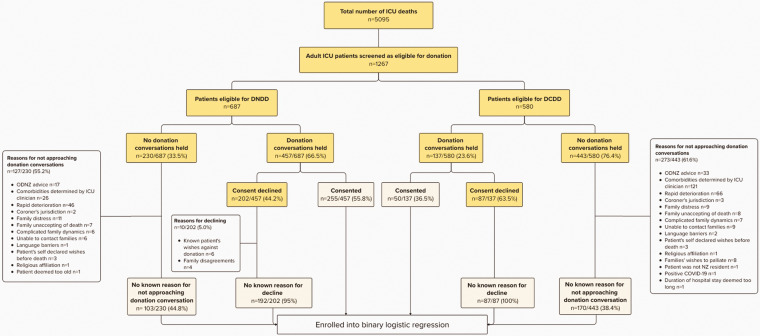
Incidences of donation conversations and outcomes.

A total of 594/1267 cases (46.9%) were approached for donation conversations, with more by the DNDD pathway (457/594, 76.9%) than the DCDD pathway (137/594, 23.1%), resulting in 51.3% (305/594) consents obtained overall (Figure 1). Most conversations were led by intensive care specialists (541/594, 91.1%), followed by anaesthetists (38/594, 6.4%) and ICU nurses (15/594, 2.5%). In total, 673 eligible cases were not approached for donation conversations (DNDD 230; DCDD 443), of which 400 (59.4%) had a documented reason; however, three cases were inaccurately ruled ineligible for donation due to positive COVID-19 status, age, and prolonged hospital stay. For the 594 eligible cases that were approached for donation conversations (DNDD 457; DCDD 137), reasons for declining consent were documented for 10 DNDD cases.

### Factors associated with donation conversations and decisions

#### Likelihood of approaching donation conversations: DNDD versus DCDD

A total of 555 eligible DNDD cases were analysed for factors predicting the likelihood of donation conversations occurring (Table 2). Factors associated with an increased likelihood of conversations being approached included formally assessing and confirming brain death (*P* < 0.001) and having consultations with ODNZ (*P* = 0.004). Factors associated with a reduced likelihood of conversations being approached included the potential donor patient being Māori (*P* = 0.002) or being admitted into the ICU with encephalopathy (*P* = 0.011), cardiac failure/cardiovascular disease (*P* < 0.001), and cardiac arrest (*P* = 0.003).

**Table 2.** Model predicting likelihood of conversations for donation by neurological determination of death using regression analysis.

Variables	Beta coefficient	Significance (*P* value)	Odds ratio	95% confidence interval	
				Lower	Upper
(Intercept)	1.463	0.158	4.32	0.568	32.861
Age, years					
81+	18.787	1	144277618	0	.^a^
61–80	−1.462	0.09	0.232	0.043	1.259
41–60	−0.597	0.475	0.55	0.107	2.832
21–40	−0.991	0.214	0.371	0.078	1.774
15–20	0^a^	.	1	.	.
Gender					
Men	0.178	0.588	1.195	0.627	2.28
Women	0^a^	.	1	.	.
Ethnicity					
Asian	−0.418	0.528	0.658	0.18	2.411
Pasifika	−0.495	0.371	0.609	0.206	1.806
Māori	−1.135	0.002	0.321	0.156	0.664
New Zealand European	0^a^	.	1	.	.
Year of death					
During COVID-19	−0.5	0.108	0.606	0.33	1.115
Not during COVID-19	0^a^	.	1	.	.
Reason for admission					
Homicide	−0.242	0.87	0.785	0.043	14.266
Suicide	0.354	0.597	1.425	0.383	5.297
Cancer	20.631	0.999	911672602	0	.a
Trauma other	−0.425	0.646	0.654	0.107	4.014
Trauma road	−0.319	0.577	0.727	0.237	2.227
Encephalopathy	−1.929	0.011	0.145	0.033	0.647
Cardiac failure/cardiovascular disease	−3.517	<.001	0.03	0.004	0.204
Cardiac arrest	−1.612	0.003	0.2	0.07	0.57
Brain ischaemia	−0.827	0.081	0.437	0.173	1.106
Brain haemorrhage	0^a^	.	1	.	.
Intensive care unit length of stay, days					
8+	−0.297	0.725	0.743	0.142	3.888
4–7	0.333	0.59	1.395	0.415	4.687
0–3	0^a^	.	1	.	.
Intensive care unit level					
Three	0.681	0.166	1.977	0.755	5.179
Two	−0.293	0.597	0.746	0.253	2.205
One	0^a^	.	1	.	.
Consulted with Organ Donation New Zealand					
Yes	0.924	0.004	2.52	1.335	4.756
No	0^a^	.	1	.	.
Confirmed brain death					
Yes	2.951	<.001	19.13	8.438	43.369
No	0^a^	.	1	.	.

a Inestimable due to insufficient variability within category.

A total of 302 eligible DCDD cases were analysed (Table 3). Again, consulting the case with ODNZ was significantly associated with an increased likelihood of approaching DCDD conversations (*P* < 0.001), while reasons for ICU admission including respiratory failure (*P* < 0.001), gastrointestinal failure (*P* = 0.002), sepsis (*P* = 0.013), suicide (*P* = 0.03), among others, were linked to a reduced likelihood of conversations being approached.

**Table 3.** Model predicting likelihood of conversations for donation by circulatory determination of death using regression analysis.

Variables	Beta coefficient	Significance (*P* value)	Odds ratio	95% confidence interval	
				Lower	Upper
(Intercept)	−0.749	0.569	0.473	0.036	6.235
Age, years					
61–70	−0.922	0.321	0.398	0.064	2.458
41–60	0.067	0.938	1.069	0.196	5.845
21–40	0.314	0.721	1.368	0.244	7.662
15–20	0^a^	.	1	.	.
Gender					
Men	0.499	0.213	1.648	0.751	3.614
Women	0^a^	.	1	.	.
Ethnicity					
Other	−0.83	0.502	0.436	0.039	4.921
Asian	−1.016	0.121	0.362	0.1	1.308
Pasifika	−1.002	0.17	0.367	0.088	1.535
Māori	0.293	0.507	1.34	0.564	3.186
New Zealand European	0^a^	.	1	.	.
Year of death					
During COVID-19	0.15	0.678	1.162	0.572	2.363
Not during COVID-19	0^a^	.	1	.	.
Reason for admission					
Respiratory failure	−4.74	<0.001	0.009	0.001	0.106
Gastrointestinal failure	−4.057	0.002	0.017	0.001	0.213
Sepsis	−2.364	0.013	0.094	0.014	0.61
Suicide	−1.566	0.03	0.209	0.051	0.86
Cancer	−3.744	0.014	0.024	0.001	0.472
Trauma other	−0.595	0.434	0.552	0.124	2.451
Trauma road	−1.857	0.025	0.156	0.031	0.796
Encephalopathy	−0.86	0.345	0.423	0.071	2.519
Cardiac failure/cardiovascular disease	−2.676	<0.001	0.069	0.02	0.235
Cardiac arrest	−2.145	<0.001	0.117	0.035	0.392
Brain ischaemia	−1.701	0.017	0.183	0.045	0.734
Brain haemorrhage	0^a^	.	1	.	.
Intensive care unit length of stay, days					
8+	−0.725	0.209	0.484	0.156	1.502
4–7	−0.592	0.218	0.553	0.216	1.419
0–3	0^a^	.	1	.	.
Intensive care unit level					
Three	0.312	0.812	1.366	0.105	17.761
Two	−0.341	0.731	0.711	0.101	4.994
One	0^a^	.	1	.	.
Hospital accredited for donation through circulatory determination					
Yes	1.868	0.099	6.478	0.705	59.521
No	0^a^	.	1	.	.
Consulted with Organ Donation New Zealand					
Yes	2.019	<0.001	7.528	3.545	15.988
No	0^a^	.	1	.	.

a Inestimable due to insufficient variability within category.

#### Likelihood of obtaining consent: DNDD versus DCDD

A total of 443 approached DNDD cases were analysed (Table 4). Factors linked to an increased likelihood of families consenting to organ donation were formal confirmation of brain death (*P* < 0.001) and consultation with ODNZ (*P* < 0.001), prior to approaching families. Homicide also showed an association with increases in consent likelihood (*P* = 0.014); however, the wide 95% confidence intervals (1.732 to 134.448) indicate uncertainty in magnitude. Factors associated with a reduced likelihood of families consenting were potential donor patients being Māori (*P* < 0.001), Pasifika (*P* < 0.001), and Asian (*P* = 0.004).

**Table 4.** Model predicting likelihood of obtaining consent for donation by neurological determination of death using regression analysis.

Variables	Beta coefficient	Significance (*P* value)	Odds ratio	95% confidence interval	
				Lower	Upper
(Intercept)	−3.676	<0.001	0.025	0.003	0.21
Age, years					
81+	0.351	0.8	1.42	0.094	21.378
61–80	0.446	0.484	1.562	0.448	5.449
41–60	0.067	0.908	1.069	0.343	3.331
21–40	0.315	0.581	1.37	0.448	4.192
15–20	0^a^	.	1	.	.
Gender					
Men	−0.459	0.123	0.632	0.353	1.132
Women	0^a^	.	1	.	.
Ethnicity					
Asian	−1.298	0.004	0.273	0.113	0.66
Pasifika	−2.173	<0.001	0.114	0.048	0.271
Māori	−2.099	<0.001	0.123	0.057	0.262
New Zealand European	0^a^	.	1	.	.
Year of death					
During COVID–19	0.058	0.835	1.06	0.613	1.832
Not during COVID–19	0^a^	.	1	.	.
Reason for admission					
Homicide	2.725	0.014	15.26	1.732	134.448
Suicide	0.778	0.131	2.177	0.793	5.979
Cancer	1.694	0.235	5.443	0.332	89.374
Trauma other	1.137	0.091	3.118	0.835	11.645
Trauma road	0.558	0.295	1.747	0.615	4.962
Encephalopathy	0.492	0.548	1.635	0.329	8.124
Cardiac failure/cardiovascular disease	0.022	0.982	1.022	0.143	7.334
Cardiac arrest	0.212	0.734	1.236	0.365	4.181
Brain ischaemia	−0.136	0.764	0.873	0.359	2.12
Brain haemorrhage	0^a^	.	1	.	.
Intensive care unit length of stay, days					
8+	−0.225	0.789	0.799	0.154	4.139
4–7	0.481	0.339	1.617	0.603	4.334
0–3	0^a^	.	1	.	.
Intensive care unit level					
Three	−0.197	0.752	0.822	0.242	2.786
Two	0.692	0.35	1.998	0.469	8.522
One	0^a^	.	1	.	.
Consulted with Organ Donation New Zealand					
Yes	3.493	<0.001	32.883	9.542	113.323
No	0^a^	.	1	.	.
Confirmed brain death					
Yes	1.939	<0.001	6.951	3.607	13.394
No	0^a^	.	1	.	.
Who held donation conversation					
Anaesthetist	1.316	0.068	3.727	0.909	15.283
Nurse	−0.096	0.9	0.908	0.202	4.087
Intensive care specialist	0^a^	.	1	.	.

a Inestimable due to insufficient variability within category.

A total of 134 approached DCDD cases were analysed (Table 5). Consultation with ODNZ prior to approaching families (*P* = 0.002), was a factor associated with increases in consent likelihood. Additionally, factors including the patient being a man (*P* = 0.019) and an ICU LOS of 4 to 7 days (*P* = 0.003) were also associated with increased consent likelihood, while families of Māori patients were associated with decreased consent likelihood (*P* = 0.002).

**Table 5.** Model predicting likelihood of obtaining consent for DCDD using regression analysis.

Variables	Beta coefficient	Significance (*P* value)	Odds ratio	95% confidence interval	
				Lower	Upper
(Intercept)	−0.623	0.835	0.536	0.002	190.059
Age, years					
61–70	−1.511	0.512	0.221	0.002	20.087
41–60	−1.758	0.433	0.172	0.002	13.939
21–40	−1.443	0.489	0.236	0.004	14.058
15–20	0^a^	.	1	.	.
Gender					
Men	1.81	0.019	6.108	1.344	27.756
Women	0^a^	.	1	.	.
Ethnicity					
Other	−21.862	1	<.001	0	.a
Asian	−2.526	0.095	0.08	0.004	1.556
Pasifika	−1.948	0.234	0.143	0.006	3.532
Māori	−3.551	0.002	0.029	0.003	0.275
New Zealand European	0^a^	.	1	.	.
Year of death					
During COVID–19	−1.274	0.06	0.28	0.074	1.053
Not during COVID–19	0^a^	.	1	.	.
Reason for admission					
Respiratory failure	19.722	1	367,587,934	0	.a
Gastrointestinal failure	22.106	1	3,986,038,779	0	.a
Sepsis	−1.299	0.438	0.273	0.01	7.282
Suicide	0.376	0.711	1.456	0.2	10.586
Cancer	24.248	1	3.3957 × 10^10^	0	.a
Trauma other	−1.02	0.365	0.361	0.04	3.28
Trauma road	−1.025	0.803	0.359	0	1138.243
Encephalopathy	0.376	0.806	1.457	0.072	29.476
Cardiac failure/ cardiovascular disease	−0.951	0.45	0.386	0.033	4.554
Cardiac arrest	0.329	0.749	1.39	0.186	10.393
Brain ischaemia	−1.064	0.395	0.345	0.03	4
Brain haemorrhage	0^a^	.	1	.	.
Intensive care unit length of stay, days					
8+	1.759	0.118	5.806	0.641	52.563
4–7	2.716	0.003	15.123	2.52	90.754
0–3	0^a^	.	1	.	.
Intensive care unit level					
Three	−1.213	0.472	0.297	0.011	8.114
Two	−1.999	0.26	0.135	0.004	4.38
One	0^a^	.	1	.	.
Hospital accredited for donation through circulatory determination					
Yes	0.216	0.902	1.241	0.04	38.221
No	0^a^	.	1	.	.
Consulted with ODNZ					
Yes	2.969	0.002	19.476	3.047	124.503
No	0^a^	.	1	.	.
Who held donation conversation					
Anaesthetist	−0.674	0.568	0.51	0.05	5.154
Nurse	21.7	1	2654719666	0	.a
Intensive care specialist	0^a^	.	1	.	.

a Inestimable due to insufficient variability within category.

## Discussion

### Summary of key findings

This analysis of eligible donor patients in NZ adult ICUs identified low rates of conversation approaches and consent. Factors such as ethnicity, gender, ICU LOS, and reason for admission showed some association. Two potentially modifiable factors associated with an increased likelihood of approaching conversations and obtaining family consent were early consultation with ODNZ and timely brain death confirmation (for DNDD).

### Relationship to previous studies

This study found an overall donation conversation approach rate of 46.9% in NZ, resulting in a consent rate of 51.3%, both of which are lower than international reports—Australia: 93.8% approach rate and 55% consent rate overall,^16^ UK: 72% consent rate through DNDD,^4^ Spain: 88.4% consent rate overall.^1^ New Zealand operates under an ‘opt-in’ donation system, while countries such as Spain implement an ‘opt-out’ or ‘presumed consent’ framework,^6^ which could influence consent rates. However, Australia also utilises an opt-in model, and reports higher consent rates; while conversely, many countries have observed no significant change in consent rates following the adoption of an ‘opt-out’ legislative approach.^6^ Rather than advocating for a shift to an opt-out model, the Australian and New Zealand Intensive Care Society (ANZICS) emphasises the importance of compassionate communication to facilitate informed and enduring decisions by families.^6^ Additionally, the absence of a donor registry in New Zealand, in contrast to other jurisdictions such as Australia,^6^ may contribute to lower consent rates. Establishing a donor register accessible by clinicians could help communicate recorded information to families thereby supporting donation decisions during end-of-life discussions.

The characteristics of eligible organ donation patients in NZ ICUs were consistent with studies from Canada,^9^ the US,^10^ and the UK,^11^ showing similar demographics: predominantly middle-aged, European, admitted into tertiary centres, for less than 3 days, with more men than women, and common admissions due to brain haemorrhage/ischaemia, cardiac arrest or trauma. Similarly, the Canadian study^9^ found no significant link between healthcare centre size and family decisions.

A comparative study examining survey results from 19 organ donation agencies in the US, conducted before and during the COVID-19 pandemic, revealed a significant decrease in donor consent rates.^17^ The study highlighted a reduction in the on-site presence of donation agency staff and an increased hesitancy among families to consent to organ donation during the pandemic, citing emotional stress, communication barriers, and fears related to COVID-19 as key factors influencing their decisions. Interestingly, there was no significant association between the COVID-19 pandemic and conversation or consent rates for organ donation in New Zealand. This may be attributed to the timeline of government response activities,^18^ which demonstrated the implementation of an early, robust public health strategy, including strict border controls and lockdowns to manage viral transmissions. Proactive communication and timely updates between health authorities and the community were maintained, likely fostering public trust in the healthcare system and reducing anxieties associated with the pandemic.^18^ Additionally, ODNZ maintained a strong educational presence during this period, ensuring ongoing engagement and advocacy for donation initiatives.^5^

A positive association between LOS of 4r to 7 days, male gender and DCDD consent is consistent with findings in the US^19^ and UK,^11^ where families of male patients were also more likely to consent. A paediatric study^20^ suggested that ICU LOS is correlated to an increased risk of infection, prompting treatment plan decisions and donation conversations within 5 days.^21,22^ Rao et al.^23^ further described that the time needed to assess withdrawal of active treatment and the likelihood of asystole to meet DCDD criteria may explain the positive association between an ICU LOS of several days and DCDD consent.

In NZ, families of non-Caucasian patients were linked to a reduced likelihood of providing consent. Studies investigating factors associated with consent rates also found similar trends for Australia^24^ the US^10^ and the UK,^11^ while a study investigating obstacles to donation cited minority ethnic groups expressing reluctance due to lack of organ donation/transplant awareness, distrust of the medical industry, or fear of premature death.^25^ Cultural beliefs or bereavement customs may also influence these differences. In eastern cultures, Confucian values emphasise home death and whole-body burials,^26–28^ while Māori traditions in NZ, such as tangihanga, involve sacred mourning rituals at a marae and burial.^29^ Studies on Māori views of organ donation^30,31^ highlight concepts of interconnectedness, altruism and reciprocity, where anonymous donation may conflict without appropriate support. Pasifika cultures also place strong values on spirituality and stoicism,^32,33^ which may influence attitudes towards organ donation conversations during bereavement.

Although cultural beliefs may not inherently oppose donation for families, they may necessitate culturally sensitive approaches from ICU staff to reconcile the concept of death,^34–36^ particularly when mechanical ventilation maintains the appearance of life.^36^ ICU staff often report discomfort initiating donation conversations with families who wish to take their loved one home or perform whole-body rituals.^37^ There is also apprehension about adding burden, especially when families struggle to understand neurological death.^37–39^ This context may elucidate why non-Caucasian families in NZ were also associated with a reduced likelihood of being approached for donation discussions.

Admission reasons, including encephalopathy, cardiovascular disease, and respiratory failure, were associated with a reduced likelihood of donation conversations occurring but did not impact family consent. Factors related to patient diagnoses may be considered non-modifiable in the patient; however, determining whether these factors deem a patient ineligible for organ donation may be subject to the attending clinician's interpretation of their significance and may, to some extent, be modifiable. Studies suggest ICU staff may struggle to recognise donation eligibility in non-haemorrhagic or non–cardiac arrest cases.^8,40^ Moreover, staff engagement in donation conversations with family is reportedly influenced by their confidence in explaining both the clinical pathophysiology and donation processes leading to DNDD and DCDD.^41–43^ Routine conversation simulations have been shown to improve staff confidence, understanding, and morale, enhancing conversation rates and family support.^42,44^

Furthermore, increasing referral rates to organ donation agencies and engaging in early consultations are modifiable aspects of ICU practice. These initiatives can be integrated into routine end-of-life protocols supported by effective referral reminders^4^ and have been shown to improve the outcomes of donation conversations.^4,11,45–47^ Involving organ donation specialists early through telephone^45^ or collaborative discussions^46,47^ has enabled staff to recognise patient eligibility, receive diagnostic and conversational advice and improve communicational skills.

ODNZ actively promotes referrals for patients who may have potential contraindications to ensure that potential donors are not prematurely ruled out and to address misconceptions regarding their eligibility,^12^ such as the cases in this study deemed ineligible due to COVID-19 status, age, and ICU LOS. It is also essential to consider whether the cohort of patients who were not approached for donation discussions (DNDD: 26, DCDD: 121)—citing ‘comorbidities determined by the ICU clinician’ as the reason (Figure 1)—could have been reconsidered for donation through a referral to ODNZ.

Additionally, among the 289 families who declined, the reasons for their decisions were documented for only 10. As families are not obligated to provide a rationale for their choices, comprehensive data on this aspect are lacking. Nonetheless, it is important to consider whether modifiable factors, such as early ODNZ referrals and donation specialist involvement, might have influenced these outcomes differently. According to the potential donor audit conducted in the UK in 2024,^48^ referral rates sent to organ donation organisations reached 99% for eligible DNDD cases and 93% for DCDD cases. Given the 47.1% consultation rate with ODNZ, it is reasonable to assert that NZ has the opportunity to adopt similar initiatives designed to enhance referral rates, which could, in turn, improve both conversation approach rates and consent rates.

A lower rate of formal brain death testing and subsequent brain death confirmation at 53% was also seen across NZ ICUs for DNDD-eligible patients. In contrast, recent UK reports highlighted that 76% of 2029 eligible DNDD patients had brain death testing and confirmation.^49^ Timely brain death confirmation for DNDD, prior to approaching families, is arguably a modifiable aspect in ICU practice as it supports ICU staff and families in transitioning from curative to end-of-life care, thereby facilitating donation discussions.^4,11,39^

Kananeh et al.^10^ identified that completing the apnoea test as part of brain death confirmation indicated 3.7 times higher rates for consent, while Beigzadeh et al.^49^ found that having brain death confirmed or witnessed by family aided understanding and donation decision making. Angiograms have been suggested to expedite confirmation;^50^ however, rural facilities may lack readily available radiologists and equipment and costs may be prohibitive in publicly funded systems^51^ such as NZ. Furthermore, 16 NZ ICUs also lack guaranteed 24-hour ICU specialist availability onsite,^13^ potentially delaying structured brain death testing for donation conversations.

The ANZICS statement on death^6^ addresses the importance of prompt testing, protocol adherence for assessments, and training around brain death testing to minimise uncertainty for families and support staff competence. For Australia, referrals to organ donation staff are triggered when there is a medical consensus that a patient is at the end of life, which then prompts assessments to confirm neurological deaths alongside other investigations to determine donation suitability.^6^ NZ could benefit from implementing similar referral triggers and prompts for healthcare providers, potentially enhancing approaches to donation conversations and improving consent rates. Furthermore, ANZICS recommends standardised training programmes to ensure staff remain familiar and confident with the procedures involved in responding to irreversible brain injuries and determining brain death, incorporating a multidisciplinary approach in the assessment processes and training.^6^

### Limitations and strengths

The retrospective design may have been limiting due to a lack of quality control over data availability and inconsistency, leading to the exclusion of some variables from the analysis. Despite rigorous testing for model fit and data quality, the nature of clinical data distribution may have affected interpretability. These data are from one country only and focus on the adult ICU setting, therefore may not be generalisable to other healthcare systems or units such as the emergency department or paediatric intensive care.

However, study strengths include being the first retrospective analysis of factors influencing donation conversations and decisions in NZ, with no missing values for included variables. The use of a central database allowed time and cost efficiency, access to data from all adult ICUs in the country, and a large sample size, while additional evaluation by analysts enhanced the credibility of the findings.

### Implications

The results of this study reveal low rates of conversation approaches, consent, ODNZ referrals, and brain death confirmation in NZ compared with international reports. These findings highlight the need for enhanced staff training and programme improvements, particularly emphasising the importance of early consultation with ODNZ and timely brain death confirmation to facilitate organ donation more effectively. The current national data registry could be improved to record details routinely on the role of other healthcare professionals involved in donation conversations, which is currently inconsistent or absent in the registry, making it difficult to assess collaborative approaches, unlike overseas systems.^45,46,52^ Furthermore, international systems also record patients’ known donation wishes, staff training levels, conversation frequency,^53,54^ and timing at key stages; before, between, and after two brain death tests,^55^ enabling broader analysis. Expanding ethnicity categories for patients and families could also improve understanding of cultural needs. Future research could involve qualitative designs exploring staff and families’ perspectives on organ donation, and diverse ethnic groups in NZ, to understand decision-making factors better.

## Conclusion

In summary, this study underscores the significant gaps in donation conversations, consent, ODNZ referrals, and brain death confirmation rates among potential donor patients in NZ ICUs when compared to international reports. Factors influencing donation conversations included ethnicity, gender, ICU LOS, and reason for ICU admission. Early consultation with ODNZ and timely brain death confirmation were associated positively with conversation rates and consent rates and were considered modifiable factors. The inclusion of additional variables to the national data registry would enhance its utility, and future research to explore the experience of staff and families regarding organ donation would help to understand decision making processes.

## Supplemental Material

sj-pdf-1-aic-10.1177_0310057X251357317 - Supplemental material for A retrospective analysis of patients eligible for organ donation in adult intensive care units in Aotearoa New Zealand Supplemental material, sj-pdf-1-aic-10.1177_0310057X251357317 for A retrospective analysis of patients eligible for organ donation in adult intensive care units in Aotearoa New Zealand by Lydia Shim, Cynthia J Wensley and Rachael L Parke in Anaesthesia and Intensive Care

## Data Availability

Restrictions apply to the availability of these data, which were used under licence for this study. Data may be available on reasonable request with the permission of Organ Donation New Zealand.
