# Supplementary material for: A retrospective analysis of patients eligible for organ donation in adult intensive care units in Aotearoa New Zealand
Source: Anaesth Intensive Care. 2025 Aug 14;54(1):18–30. doi: 10.1177/0310057X251357317 (PMC12779765; doi:10.1177/0310057X251357317)
Supplement: sj-pdf-1-aic-10.1177_0310057X251357317 - Supplemental material for A retrospective analysis of patients eligible for organ donation in adult intensive care units in Aotearoa New Zealand [file sj-pdf-1-aic-10.1177_0310057X251357317.pdf]

Supplementary file 1

Labels for reasons for admission and their diagnoses.

**Reasons for admission**	**Diagnoses included:**
Brain haemorrhage	• Subarachnoid haemorrhage<br>• Intracerebral haemorrhage<br>• Intraparenchymal haemorrhage<br>• Aneurysm ruptures<br>• Brainstem haemorrhage
Brain ischaemia	• Cerebral infarct/embolism<br>• Metabolic coma<br>• Spinal cord compression<br>• Seizures
Encephalopathy	• Microbial encephalopathy<br>• Hypoxic ischaemic encephalopathy
Cancer	• Tumours<br>• Haematological cancers<br>• All other forms of cancer
Cardiac arrest	• Out-of-hospital cardiac arrest<br>• Pulseless electrical activity arrest
Cardiac failure/cardiovascular disease	• Congestive heart failure<br>• Aortic dissection<br>• Cardiomyopathy<br>• Myocardial infarct<br>• Cardiogenic shock/hypotension<br>• Rupture varicose veins
Sepsis	• Bacteraemia<br>• Necrotising fasciitis<br>• Septic shock
Suicide	-
Homicide	-
Road trauma	• Motor vehicle traffic accidents
Other trauma	• Falls<br>• Collisions<br>• Accidental injuries

Multi-organ failure	• Two or more solid organ failures without sepsis being the primary cause
Gastrointestinal failure	• Large and small intestinal ischaemia/obstruction/perforation
Respiratory failure	• Exacerbated chronic airflow limitation<br>• Exacerbated asthma<br>• Cystic fibrosis
Autoimmune disease	• Guillain-Barré syndrome<br>• Adult-onset Still's disease<br>• Macrophage activation syndrome
